# Describing the factors that influence the process of making a shared-agenda in Japanese family physician consultations: a qualitative study

**DOI:** 10.1186/s12930-015-0023-6

**Published:** 2015-06-05

**Authors:** Michiko Goto, Shoji Yokoya, Yousuke Takemura, Alberto Alexander Gayle, Tsukasa Tsuda

**Affiliations:** Department of Education and Research in Family and Community Medicine, Mie University Graduate School of Medicine, 2-174 Edobashi, Tsu, Mie 514-8507 Japan; Community-based Medicine Education Station kitaibaraki, Faculty of Medicine, University of Tsukuba, 1-1-1 Tennodai, Tsukuba, Ibaraki 305-8575 Japan; Department of Family Medicine, Mie University School of Medicine & Graduate School of Medicine, 2-174 Edobashi, Tsu, Mie 514-8507 Japan; Center for Medical and Nursing Education, Mie University School of Medicine, 2-174 Edobashi, Tsu, Mie 514-8507 Japan; Kikugawa municipal Family Medicine Center, 1055-1 Akatsuti, Kikugawa, Shizuoka 439-1507 Japan

**Keywords:** Agenda-sharing, Communication, Doctor-patient relationship, Patient’s explanatory model, Taking a medical history

## Abstract

**Background:**

Patients cannot always share all necessary relevant information with doctors during medical consultations. Regardless, in order to ensure the best quality consultation and care, it is imperative that a doctor clearly understands each patient’s agenda.

The purpose of this study was to analyze the process of developing a shared-agenda during family physician consultations in Japan.

**Methods:**

We interviewed 15 first time patients visiting the outpatient clinic of the Department of Family Medicine in the hospital chosen for the investigation, and the 8 family physicians who examined them. In total we observed 16 consultations. We analyzed both patients’ and doctors’ narratives using a modified grounded theory approach.

**Results:**

For patients, we found four main factors that influenced the process of making a shared-agenda: past medical experiences, undisclosed but relevant information, relationship with the family physician, and the patient’s own explanatory model. In addition, we found five factors that influenced the shared agenda making process for family physicians: understanding the patient’s explanatory model, constructing the patient-doctor relationship, physical examination centered around the patient’s explanatory model, discussion-styled explanation, and self-reflection on action.

**Conclusions:**

The findings suggest that patient satisfaction would be increased if family physicians are proactive in considering these factors with respect to both the patient’s agenda, and their own.

## Background

Anecdotal evidence suggests that when patients visit a doctor, they typically have an agenda to discuss [1–3]. The agenda includes particular problems which patients want to discuss with their doctor; for example: patients’ ideas about the reasons of their sickness, seriousness of a symptom, recuperation of the problems, expectations regarding the course of the illness or prognosis, medical examinations and prescription, referral to a specialist and explanation for an absence from school or work [4, 5].

On the other hand, doctors also have an agenda, which typically revolves around diagnosis and management. However, taking the scope and dimensions of their work into consideration, family physicians, must include broader considerations [2].

Levenstein noted that it is important to combine the agendas from both patient and doctor [6]. Moreover, the literature suggests that doctors may frequently fail to fully understand patients’ true expectations and requests [2, 7]. Or alternatively, patients may not be able to share their agenda completely with their doctors [8]. In such case, his unspoken agenda is referred to as the patient’s hidden agenda [9].

Marple et al. suggest that a mismatch of agendas between patients and doctors lowers patients’ satisfaction [5]. Other research suggests that agenda mismatching makes compliance lower and may affect the outcome [10–12]. Indeed, one of the reasons that some patients repeatedly go “doctor shopping” may be because of agenda mismatching [13]. All things considered, it is clear that the ability for patients to share their agendas well potentially lowers costs and reduces consultation times [14, 15].

Charles et al. described the process of agenda-sharing with the expression “it takes two to tango” [16]. Accordingly, the factors that prevent or promote agenda-sharing should be defined in order to make agenda-sharing more consistent and productive in terms of patient outcomes and consultation costs. However, until recently, there has been little research evidence about the factors which affect the agenda-sharing process.

Thus, the purpose of this research is to describe the factors that influence the process of making a shared-agenda in Japanese family physician consultations, by analyzing patient and doctor narratives.

This study was approved by the Research Ethics Committee of the Faculty of Medicine at Mie University.

## Methods

### Sampling and recruitment

Prior to recruiting patients, we obtained informed, verbal and written consent from all staff members of the Department of Family Medicine in the hospital chosen for the investigation to observe their patient consultations. Patients were then recruited while waiting to be examined by the consenting physicians (n = 8). We screened patients and asked those who were first-time visitors, to participate in our research after explaining the research purpose, both verbally and in writing. From among 50 new patients screened, 15 finally consented to participate in this research.

#### Data collection

Patients and doctors met in standard consultation rooms, and a member of our research team observed the consultation, and took written notes, but did not participate in any way. After the initial consultation, the researcher led the patient to a separate interview room to conduct an interview in private. Researchers then interviewed the doctor, generally later that day, when the clinic had finished.

Semi-structured interviews were conducted to avoid using leading questions, with the aim of eliminating any potential for researcher bias [17]. Supplementary questions that aimed to clarify respective answers were posed, as needed, during the main questioning in a way that did not interrupt the flow of the interviews (Table 1). The questions were selected following a thorough review of previous studies concerning the communication gap between doctors and patients, and after discussing with other members.

**Table 1:** Questionnaire (Questions used in post-consultation interviews) Questions toward patients

Questions toward patients	
1	What do you think about your own illness?
2	What did you want to convey to your doctor most? Were you able to convey it?
3	What do you think the reason was for your in/ability to express yourself during the consultation? Were you satisfied with yourself?
4	Can you describe your ideal doctor?
Question toward doctors	
1	How did you listen to the patient?
2	What do you think the patient wanted to say the most?
3	What did you emphasize most in the consultation?
4	Do you think the patient was satisfied?

All researcher-patient and researcher-doctor interviews were audio recorded with consent from both the patient and doctor. Audio recordings were sent to a third party for transcription, which was later used for analysis. The written notes from the doctor-patient consultation were also collected, to allow for comparison between patient and doctor recollections of the consultation with the actual events. All samples so obtained were coded to maintain anonymity.

### Analysis

**Fig. 1:**
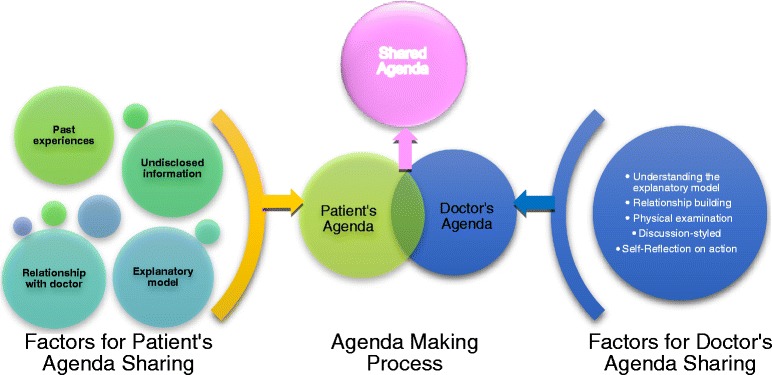
Overview of shared-agenda making process

For the analysis, a modified grounded theory approach was used [18] wherein two researchers categorized the data, and constructed a final theory together with another researcher (Fig. 1). In cases of uncertainty, the decision of the researcher who conducted the interview was given priority.

## Results

Overall, interviews lasted 30 to 40 min for patients, and 15 to 20 min for doctors. The interview was terminated once the conversation seemed to have reached theoretical saturation—in other words, when similar opinions had been repeatedly heard and no more new opinions were likely to be heard [19]. The subjects were 15 patients, and 8 doctors; in total, 16 consultations were observed. The doctors consisted of 5 male and 3 female and the patients consisted of 11 female and 4 male (Table 2).

**Table 2:** Consultation information

Doctor information			Case information	Patient information			
Doctor ID	Sex	Years in practice	Chief complaint	Patient ID	Age	Sex	Occupation
D1	Female	4	The diarrhea caused by bacterial infection	M1	69	Male	Retired
D2	Male	2	Throbbing of the chest, Irritation, Depression	F1	59	Female	Housewives
D3	Female	4	Hypertension	F2	51	Female	Office worker
D1	Female	4	Routine screening for breast cancer, Numbness of joints	F3	56	Female	Independent business
D4	Female	4	Swelling of the finger	F4	46	Female	Teacher
D4	Female	4	Phlegm obstructing the throat	F5	63	Female	Housewives
D5	Male	17	Caisson disease	F6	25	Female	Medical representative
D6	Male	3	Stomachache	M7	49	Male	Policeman
D4	Female	4	Increased urinary frequency	M8	36	Male	Factory worker
D7	Male	12	Venous inflammation of the lower leg, Collagen disease	F9	53	Female	Housewives
D6	Male	3	Abnormal swelling of the lower body	F10	50	Female	Housewives
D5	Male	17	Nausea, “strange feeling” in stomach, listlessness	M11	26	Male	Builder
D5	Male	17	Brain disease, exhausting	F12	20	Female	Nursing student
D2/D7	Male/male	2/12	Hypertension; unusual findings on examination	F13	53	Female	Worker
D8	Male	34	Gastric cancer, gastric ulcer	F14	68	Female	Housewives

The patient’s statements were grouped into four categories of factors affecting the agenda-sharing process:

1. Past medical experiences
2. Undisclosed but relevant information
3. Relationship with the family physician
4. Patient’s own explanatory model

### Past medical experiences

We found that the past medical experiences of patients had an effect on their attitude towards their present doctor, which affected agenda-sharing.

In Japan’s “free access” medical system, patients are free to move between doctor and hospitals. As a result, many patients came from other clinics in hopes of better care. There were many specific reasons, such as, dissatisfaction with a previous consultation, little or no improvement in symptoms, as well as many others. In addition, several had been referred by previous doctor, who judged that they would receive superior care at a larger institution.

Accordingly, many of the patients in this study came to the hospital chosen for this investigation, due to negative experiences with previous doctors; for example.

*“The doctor said, “There’s nothing abnormal. Perhaps it’s just your imagination.” And I said, “I need medicine,” and he prescribed only Isodine and Troche. I thought it was a joke.”* (M11: male patient)

Moreover, in many cases, patients’ past medical experiences had a large impact on their attitude during medical consultations; for example:

*“I’m going to keep asking questions to my doctor as I did today.”* (M7: male patient)

In addition, while there were some patients who questioned their doctors extensively, there were others who did not want to annoy their doctors and refrained from asking any questions outside the doctors’ specialty; for example:

*“I don’t want to ask about anything outside a doctor’s specialty because I am afraid that they become annoyed with me. Asking things without considering the consequences are rude and I don’t feel comfortable doing it.”*

(F4: female patient)

### Undisclosed but relevant information

There were substantial amounts of relevant information not mentioned to the doctor.

Some of anxieties expressed by patients following the consultation were not limited to worries about the severity of their illness or the pain involved in their treatment. Patients also worried about secondary effects the disease could have on their lives, the lives of those around them, and their professional lives; for example one patient expressed concern that their employment status could be affected by their condition:

*“I can’t say that. I say that and that’s it, my job’s over, you know?”* (F13: female patient)

In addition, patients’ anxieties were also closely connected to their family background, job status and social background.

*“We did not have a father, and my mother raised me and my brother. I don’t want to make her worried.”* (M8: female patient)

This patient wanted to undergo the same treatment as his mother, who also suffered from diabetes mellitus. In his mother’s case, she remained in the hospital until her treatment was complete. Because of this, the patient seemed likely to refuse outpatient medication and seemed set on inpatient hospital care. None of these things, however, were mentioned to the doctor.

While patients demonstrated a wide range of attitudes in facing their illnesses, those patients who refrained from discussing their outlook with their doctor tended to have similar views. Several procrastinated over coming to the hospital, or ignored their disease on days where their symptoms were less noticeable. These patients talked about how they valued their time and enjoyment rather than worrying about their disease, for example, they don’t exercise even if they had been overweight, and in fact often did not give credence to medical test results that indicated more severe problems, even if symptoms were present during the medical examination.

### Relationship with the family physician

Over the course of any consultation, the patients and doctors build a relationship. During our analysis, we found there to be two general categories of relationship: viable relationships and compromised relationships. These categorizations were based on the observed level of agenda-sharing achieved as well as the patient’s own evaluation of the consultation experience.

Examples of viable relationships are characterized by the following statements:

*“He listened to me a lot, and I did not feel uncomfortable talking to him. I felt I could talk about anything with him.”* (M7: male patient)

*“He listened to me fully, and he listened to all my concerns. The doctor that I saw today was very easy to talk to.”* (F5: female patient)

*“I am very satisfied. I am glad I came. He is a great doctor.”* (F5: female patient)

On the other hand, regarding compromised relationships, many patients did not directly answer in the negative, but instead gave vague responses indicating that the communication had in fact been insufficient.

*Well, today was my first time with this doctor. I mean, I guess I talked to him.* (F4: 48 year old female patient)

Other patients clearly stated that they were not able to fully communicate with the doctor.

*“Yeah, there was something I couldn’t talk about.”* (F13: female patient)

### Patient’s own explanatory model

Each patient had their own explanatory model for describing their disease. Patients often arrived with an original explanatory model for their particular disease and situation. For example, one patient with symptoms of general fatigue worried that it could have been caused by cancer or a brain tumor, both of which had occurred in family members. Another patient who suffered from three months of diarrhea suspected an unknown type of bacteria to be the cause. A third patient, who had numbness in her hands thought that it was due to child-birth 10 years prior.

*“I feel blood is not circulating to the ends of my body. I wonder if I have this kind of abnormality because I had a baby at an older age.”* (F4: female patient)

After the first meeting with a new doctor, and hearing the doctor’s diagnosis, some patients accepted this diagnosis, while others did not. The patients who accepted the doctor’s diagnosis and integrated this information into their previous explanatory model are said to have adaptive explanatory models.

For example, one patient whose chief complaint was various pains throughout her body initially suspected it was due to decompression sickness, a condition she was aware of through her hobby of scuba diving. After the doctor’s diagnosis of the pain as stemming from job-related stress, the patient’s explanatory model changed to consider this cause as well.

*“Since such a thorough examination couldn’t find anything abnormal, I’m beginning to feel there is nothing wrong with my body.”* (F6: female patient)

Another example of an adaptive explanatory model is the previously mentioned, patient, who complained of general fatigue. This patient, (in her early 20s), worried that the cause of her complaint could be a brain tumor or cancer. After the doctor conducted a neurological examination, the patient felt that a CT scan, which she had initially hoped for, was no longer necessary. In this case the doctor’s diagnosis was that the fatigue was likely caused by dehydration.

*“I think I need to take better care of myself.”* (F12: female patient)

In contrast to patients who accepted the new doctor’s diagnosis, those who rejected the diagnosis are said to have a non-adaptive explanatory model. In some cases, the explanatory model previously held by the patient was re-enforced and strengthened by the conflict with the doctor’s diagnosis. In other cases, the patient seemed to lose confidence in their explanatory model while still rejecting the received diagnosis.

*“The doctor said “Don’t worry about it too much”, but that’s not going to cure my disease.”* (F12: female patient)

The doctor’s statements were grouped into five categories to clarify the factors affecting the agenda sharing process, on their part:

5. Understanding the patient’s explanatory model
6. Constructing the patient-doctor relationship
7. Physical examination centered around patient’s explanatory model
8. Discussion-styled explanation
9. Self-reflection on action

### Understanding the patient’s explanatory model

Family physicians experienced patients with widely varying levels of preparedness and understanding of their own conditions. While some patients continually postponed visits and check-ups, due to not wanting to hear bad news about their own health, other patients were steadfast when faced with even the most difficult prognosis. Patients also varied in their ability not just to accept, but to understand and deal with health problems.

*“This patient does not know how to modify his lifestyle and it may take some time before he is motivated to make any changes.”* (D4: female doctor)

Family physicians that have experience with many patients were more likely to think about their diseases, fears and uncertainties, and hopes regarding the results of the examination. These physicians take the patient’s explanatory model into consideration when announcing the results of their differential diagnosis.

*“The patient’s explanatory model is a physical disorder, and the patient requests testing. The patient has stress, but he does not think it is the cause of his illness.”*

(D5: male doctor)

### Constructing the patient- doctor relationship

The physicians included in this research were aware of the patient’s expectations, and attempted to meet these expectations in order to strengthen the doctor-patient relationship.

The physicians interviewed understood that patients had various expectations from the consultation. Some patients wished for the family physicians to understand their suffering or reassure them. Other patients hoped for, or even requested, a diagnosis that contrasted with the diagnosis received from their previous doctor, or even just a chance to complain about their previous medical experience.

*“What this patient wants from me is probably not about reducing stress.”* (D5: male doctor)

The physician’s understanding of these expectations determined the approach used during the consultation. For example, in cases where the patient felt their previous doctor had been dismissive of their fears, the physicians interviewed often felt the need to conduct a comprehensive examination in order to both gain the patient’s trust, and reestablish confidence in the medical system in general, often by conducting thorough examinations. In other cases, the physician expressed the need to calm the patient and give them assurances that they would be fine. In cases where patients had already seen several family physicians and had yet to find effective treatment, doctors assured patients that they would continue to work with the patient until an acceptable treatment was found.

Patients may react badly to a physician suggesting that their problems may be psychosomatic in nature. Doctors who sensed the potential for this often began with a physical examination to help preserve the doctor-patient relationship, before suggesting other options.

*“If I had said “Your illness is due to stress,” I think the patient would reject my diagnosis, so I purposely chose a physically-centered approach.”*

(D6: male doctor)

### Physical examination centered around the patient’s explanatory model

The physicians interviewed expressed the needed to encourage patients to understand the diagnostic process, and to show patients what they are checking for.

Family physicians assessed the appropriate strategy for the consultation given the patient’s situation, to ensure that the patient’s complaint had been fully addressed, and the patient was able to understand and talk about the diagnosis. In the case of an obese patient, the doctor planned the flow of the consultation to make sure the patient realized the problem he needed to address on his own. In another case, to dispel any concerns about neurological problems, the family physician was careful to emphasize those aspects of the examination that excluded neurological problems.

*“I tried to allocate time to talk about different things according to the patient’s symptoms.”* (D2: male doctor)

The physicians we interviewed reported using strategies to facilitate the acquisition of new explanatory models. For example, in a case where a family physician indicated to a patient that a disease was psychosomatic in nature, the patient responded with a request for a CT scan in order to check brain function. In this case, the family physician said that he made sure to do extensive nerve testing so that the patient would be convinced it was not a physical problem. This was sufficient to convince the patient that her brain function was normal without the need for a CT scan. Thus, the family physician helped to prompt the patient to come to their own conclusions and develop a new explanatory model for their disease.

*“I’m pretty sure the patient is satisfied that I performed a thorough neurological examination.”* (D5: male doctor)

### Discussion-styled explanation

Family physicians did not unilaterally announce their diagnosis, but addressed patient questions and confirmed patient’s understanding while explaining their findings.

The family physician did not simply speak, but conversed with the patient to confirm how and to what extent the patient had understood the diagnosis. In a case where a patient suffering from diarrhea asked if it might be due to some unknown fungus, the family physician did not merely reply “No, it’s not.” Instead, the family physician offered an explanation, such as the fact that a fungus alone would not cause the patient’s symptoms, and offered suggestions on how the patient might change his diet. In another case, when a patient complained about noise in their ears, the family physician named the disease and also explained how to clean the ears.

*“I told the patient not to overdo ear cleaning and not to use a hard ear pick; to loosen the tip of a commercial cotton swab because it is hard; and to clean only the shallow part instead of going too far in.”* (D1: female doctor)

In another case, after the end of the consultation, the doctor-patient connection was not over, but the family physician continued to offer help to the patient regarding her ear discomfort, and offered explanation on ear care. The family physician used an appropriate conversation for the patient’s psychosocial background and fully addressed the needs of the patient.

*“There are many cases where patients haven’t accepted a diagnosis on the first visit, so I often tell them, “Please take your time to consider this information.”*

*I thought the patient had almost prepared to accept the cure.”*

(D1: female doctor)

### Self-reflection on action

The family physician was conscious of his/her actions during the consultation.

Family physicians reflected on their consultation, and wondered how to improve, considering difficult points, areas where communication had worked well and where it hadn’t, and the timing of giving information to patients. While ordering further tests, the family physician reviewed the consultation he had just given, wondered if he had maintained the proper pace of speaking, if he had been too fast or repetitive, if he had been sufficiently specific in describing causes of the disease, if he had addressed all the worries of the patient, or if he had in fact been overly reassuring to the patient in spite of the seriousness of the disease. Through all these steps the family physician reflected on the diagnosis and consultation procedure.

*“In this case, I had information from the patient’s previous doctor, so I started asking about psychiatric problems more quickly than usual.”* (D5: male doctor)

*“I told her there is no abnormality, but that doesn’t mean she now feels relieved.”*

(D4: female doctor)

## Discussion

The respective narratives made it clear that there were factors affecting the agenda-making process from both patients and doctors. Our analysis summarized these as follows:

According to the patients’ narratives:

1. Past medical experiences
2. Undisclosed but relevant information
3. Relationship with the family physician
4. Patient’s own explanatory model

According to the doctors’ narratives:

5. Understanding the patient’s explanatory model
6. Constructing the patient-doctor relationship
7. Physical examination centered around patient’s explanatory model
8. Discussion-styled explanation
9. Self-reflection on action

This study revealed many hurdles to overcome for effective agenda-sharing between doctors and patients. Many patients were observed who had problems with their previous doctors, lacked trust in the medical system, and were hesitant to openly communicate with their new doctors. A patient’s past medical experience had lasting effects on how they communicated with their current doctor. As Dr. Iwata wrote, medical interviews are “the work of reclaiming past events and bringing them to the present [20].” For the best agenda sharing, it may be necessary for doctors to indirectly address the patient’s past unhappiness with the medical system, and allow the patient to accept the new doctor-patient relationship.

However, patients’ problems were often much more complicated than the doctors expected [21]. Not all patients shared information even when it was needed in the consultation, because of the past relationship between patient and doctor.

Therefore, during examinations, family physicians tried to draw out information from patients, such as their personality, job, as well as previous medical experiences, though not necessarily verbally. This information was used by the family physician to confirm the patient’s explanatory model, their comprehension of the disease as well as their problem solving ability. After recognizing this background, doctors considered the role expected of them from the patient. For example, through assuming a positive attitude to encourage the patient, or through trying to eliminate any distrust on the part of the patient, family physicians try to build a good doctor-patient relationship.

If a patients’ explanatory model and doctors’ diagnoses were different, the family physicians we interviewed reported using the physical examination itself to affect changes in the patient’s explanatory model. For example, in cases where patients had objected to their condition being referred to in psychosomatic terms, the physicians tended to respect the patient-held explanatory model, and consciously began with a thorough physical approach to the examination.

In this way, family physicians examining patients who face psychosocial problems were not ignoring their cues, but rather addressing these cue indirectly. While the doctors have picked up on these cues, rather than trying to force their way of thinking onto patients, through doing a thorough physical examination and objectively finding no problems, doctors hoped to allow patients to develop updated explanatory models on their own.

Previous research has found that many doctors focused solely on somatic aspects of disease and disregarded cues towards psychological, emotional or social problems. Cassell stated that doctors focus more on supporting patients’ function of internal organs than personal interests about them [22]. However, our research has shown a superficially similar yet substantially different type of interaction: while family physicians have recognized the cues to psychosomatic problems, they refrained from pushing their thinking onto patients, instead carefully carrying out the physical examination and supporting patients in developing a new explanatory model on their own.

Despite the efforts of family physicians trying to build a strong doctor-patient relationship, there were patients who did not provide doctors with the necessary information. While family physicians may pick up on some cues, there were limits to the information observation could provide. In these cases, doctors are unable to contribute to patient understanding of any new explanatory model, and ultimately the examination ended without sharing their explanatory model.

We found that a family physician’s ability of reflection-on-action [23] was one of the important factors of agenda sharing throughout the consultation process.

Through this kind of action, family physicians endeavored to create better relationship with patients. According to Cassata, active interactions make patients actively and positively participate in the interview, and more predisposed to take on more responsibility. However, in this study, we found two kinds of consultation: consultations which built viable relationships and those which built compromised relationships [24]. With the former consultation, most of patient’s agenda was understood by the family physician, and it seemed that patients disclosed their information with their family physicians more positively and felt more satisfied overall.

## Conclusion

In many cases, patients’ and doctors’ agendas were different. If doctors were aware of and understood the many factors influencing the sharing of each respective agenda, and accounted for them, they were better able to clarify patients’ real explanatory model. Ultimately, we found that such a consultation gave more satisfaction to patients and increased the possibility for a patient to accept the new explanatory model to which they are led by family physicians.

### Limitation

The number of patient interviews was 15, which is wholly sufficient for qualitative research. However, these results may not be generalizable to other primary care settings, because data collection was carried out only in the one hospital chosen for this investigation. Further research is required to address this limitation.
